# Effect of variation in objective resource value on extreme male combat in a quasi-gregarious species, *Anastatus disparis*

**DOI:** 10.1186/s12898-019-0237-9

**Published:** 2019-05-23

**Authors:** Peng-Cheng Liu, De-Jun Hao

**Affiliations:** 1 Co-Innovation Center for the Sustainable Forestry in Southern China, Nanjing Forestry University, Nanjing, Jiangsu China; 2 College of Forestry, Nanjing Forestry University, Nanjing, Jiangsu China

**Keywords:** Aggressive behaviour, Resource value, Lethal male combat, Mating status

## Abstract

**Background:**

Aggressive behaviour is widely observed in animal kingdom, which compete for resources such as territory, food and mates. Resource value is the most important non-strategic factor influencing fighting behaviour, and may vary among contests and contestants. Usually, contestants adjust their fighting behaviour when the resource value changes, and as potentially damaging and energetically costly, individuals of most species usually avoid conflict escalation. However, in a quasi-gregarious egg parasitoid, *Anastatus disparis* (Hymenoptera: Eupelmidae), mates are valuable resources and females mate only once; thus, males engage in frequently extreme combat behaviour to acquire mating opportunities, even in the absence of females. In this study, we attempted to test whether males of this species have the ability to adjust their fighting behaviour in response to changes in the objective value of female.

**Results:**

Our results suggested that objective resource value in *A. disparis* is likely to be influenced by female mating status rather than by fecundity. Consistent with a number of empirical studies, *A. disparis* males adjusted their fighting behaviour according to the value of the contested resources: males significantly increased their fighting intensity to acquire mating opportunities with virgin females but decreased their fighting intensity for mated females. We also found that rather than chemical cues, visual cues and physical sexual contact appear to play a role in determining males’ ability to detect variation in female mating status.

**Conclusions:**

Our study suggested that although in this species, males have evolved extreme fighting behaviour and females are valuable resources, males do not always escalate fighting behaviour in competition for mating with a female. Valuable resources and variation in resource value were detected and estimated by *A. disparis* males, which then adjusted their fighting behaviour accordingly and to some extent avoided incoming fighting costs.

## Background

Aggressive behaviour is widely observed in animals, which compete for resources such as food, territory and mates [1]. As potentially damaging and energetically costly, individuals of most species usually avoid conflict escalation; for example, individuals may give up before being injured [2–5]. Furthermore, attacks may not only result in damage to the receiver, but also damage to the attacker [6]. However, some species, mainly in two groups of arthropods (Insecta and Arachnoidea) [2, 7–9], show extreme fighting which the fighting end with contestants being severely injured or even killed. As predicted by Enquist and Leimar [2], the balance between the value of future resource and the value of the contested resource is an important factor determining the frequency and intensity of extreme fighting. For example, females of many arthropods mate only once or a few times, and the reproductive lifespan is short. The expected number of future matings is low or very low, and the value of current mating opportunities is high and the potential benefits of winning can exceed the costs of escalating fighting, therefore favouring extreme fighting to acquire a valuable resource [2, 4, 7, 10].

Evolutionary theory predicts that a series of factors, i.e., resource value [2, 11], the number of competitors [12, 13], relatedness [7, 14] and fighting ability [15], influence the occurrence and intensity of fights. Among that, the value of these resources is likely to be the most important non-strategic variable influencing fighting behaviour [11]. Resource value encompasses an ‘objective’ and a ‘subjective’ component [16], and usually depends on a series of factors such as quality of the resource, the expected availability of a resource in time and space [11], and the physiological state of the contestants [17]. The objective component is a property intrinsic to the resource (e.g., food size) and refers to the fitness benefits that individuals can acquire from exploiting the resource [18]. The subjective component derives from a variety of different circumstances [16] and depends on internal factors, such as the individual’s physiological state and information about the environment, independently of the resource’s objective value [11]. For example, food of a given size may have greater subjective value to hungry contestants than to satiated contestants [17]. Resource value may vary among contests and individuals for many reasons. For example, the quality or quantity of a resource may vary among contests, internal physiological states (e.g., hunger, thirst) may vary from contestant to contestant [11, 16, 17], and contestants may vary in their information about a particular resource (e.g., owner–intruder interaction) [19, 20], leading to different estimates of the value of that resource by contestants. Variation in resource value influences cost of fighting and the probability of victory [11], and a number of empirical studies have shown that animals adjust their fighting behaviour when the resource value changes [16, 21–24]. However, variation in the value of a contested resource does not always change the fighting behaviour (i.e., intensity of fighting). For instance, in male *Melittobia*, a parasitoid wasp, given the males with short lifespan and limited mating opportunity, they do not adjust their fighting behaviour when the resource value has changed, and the best strategy may be to fight whenever another male is encountered without making any assessment [25].

*Anastatus disparis* (Hymenoptera: Eupelmidae) is a quasi-gregarious egg parasitoid of several noxious species of Lepidoptera [26, 27] and shows extreme male fighting [28]. Only a few Hymenoptera species (e.g., fig wasps and some members of the genus *Melittobia*) have evolved extreme male combat behaviour [7, 29, 30]. Females are valuable resources for *A. disparis* males and mate only once, rejecting subsequent matings after they have already mated [31]; thus, males of this species show extreme fighting to acquire mating opportunities [28]. Here, we tested whether males have the ability to adjust their fighting behaviour in response to changes in the objective value of a female. The objective value of a resource may be influenced by one or more properties. For example, in this study, compared to the value of virgin females, the value of mated females is reduced to zero because males no longer acquire any mating success. In addition, many studies have shown that the resource value of female could be significantly influenced by fecundity [20, 32]. Egg loads are usually used to represent as fecundity [33], which is also correlated with female body size [34]. Therefore, we tested which property (i.e., mated status, egg loads or body size) was mainly related to resource value of females, and whether males have the ability to adjust their fighting behaviour in response to changes in the objective value of the resource. If males can detect the changes in the value, we expected that they would adjust their fighting behaviour accordingly [11, 16, 21–24], for example, by increasing fighting intensity for virgin or/and high-fecundity females with a higher resource value. Alternatively, males might not adjust their fighting behaviour because they lack the ability to detect the changes in the resource value, or consistent with the result of the study in *Melittobia* showing that males fight whenever another male is encountered without making any assessment [25]. Additionally, mating generally causes changes in sexual mating behaviour in many insects, a change that is correlated with pheromone levels [35, 36]. Assuming that males can detect the changes of female mating states and adjust their fighting behaviour accordingly, we hid a female in the arena for exploring whether chemical cues or other cues play a role in determining the male’s ability to detect female mating status.

## Results

### Lethal combat for virgin and mated females

#### Mating status

**Fig. 1:**
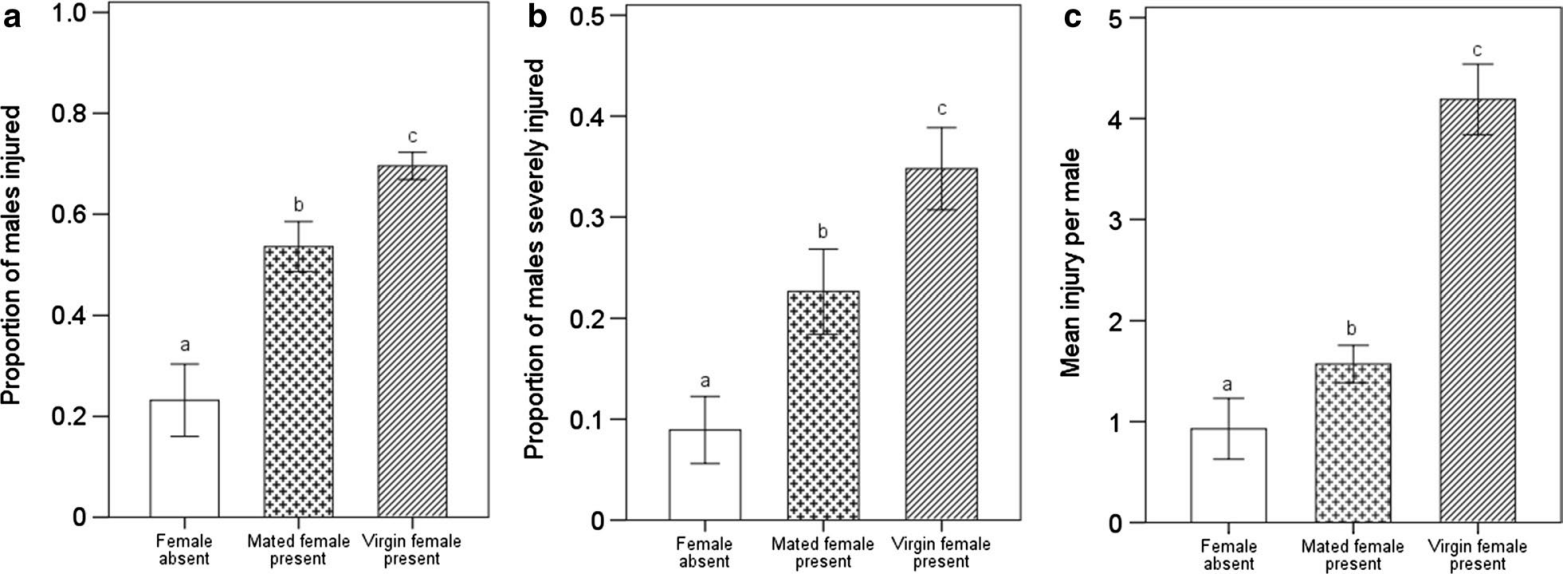
Proportions of males injured (**a**) and severely injured (**b**), and mean injury per male (**c**) after combat for 3 h under conditions of female absence, female presence (virgin and mated females). Severely injured generally involved the loss of two legs or even more serious injuries. The same letter on the column indicates no significant difference, however, different letters indicate significant difference. The error bars indicate means ± standard errors

Consistent with a previous study [28], both virgin and mated female presence significantly increased male aggression versus that in the absence of females, as measured by the proportions of males injured (Fig. 1a, virgin: *F* = 16.48, *df* = 1, *df*2 = 35, *p* < 0.001; mated female: *F* = 9.47, *df* = 1, *df*2 = 33, *p* < 0.01) and severely injured (Fig. 1b, virgin: *F* = 12.11, *df* = 1, *df*2 = 35, *p* < 0.01; mated female: *F* = 3.26, *df* = 1, *df*2 = 33, *p* < 0.05) and the mean injury per male (Fig. 1c, virgin: *F* = 12.56, *df* = 1, *df*2 = 35, *p* < 0.001; mated female: *F* = 3.07, *df* = 1, *df*2 = 33, *p* < 0.05). For acquiring mating opportunity with a virgin female, 69.57 ± 2.7% of males were injured during combat, including 34.78 ± 4.08% being severely injured. The mating status of the female had a significant effect on the males’ fighting intensity, which was higher in combat for a virgin female than for a mated female, as measured by the proportions of males injured (Fig. 1a: *F* = 6.14, *df* = 1, *df*2 = 42, *p* < 0.05) and severely injured (Fig. 1b: *F* = 4.74, *df* = 1, *df*2 = 42, *p* < 0.05) and the mean injury per male (Fig. 1c: *F* = 22.95, *df* = 1, *df*2 = 42, *p* < 0.001).

#### Female fecundity and body size

**Fig. 2:**
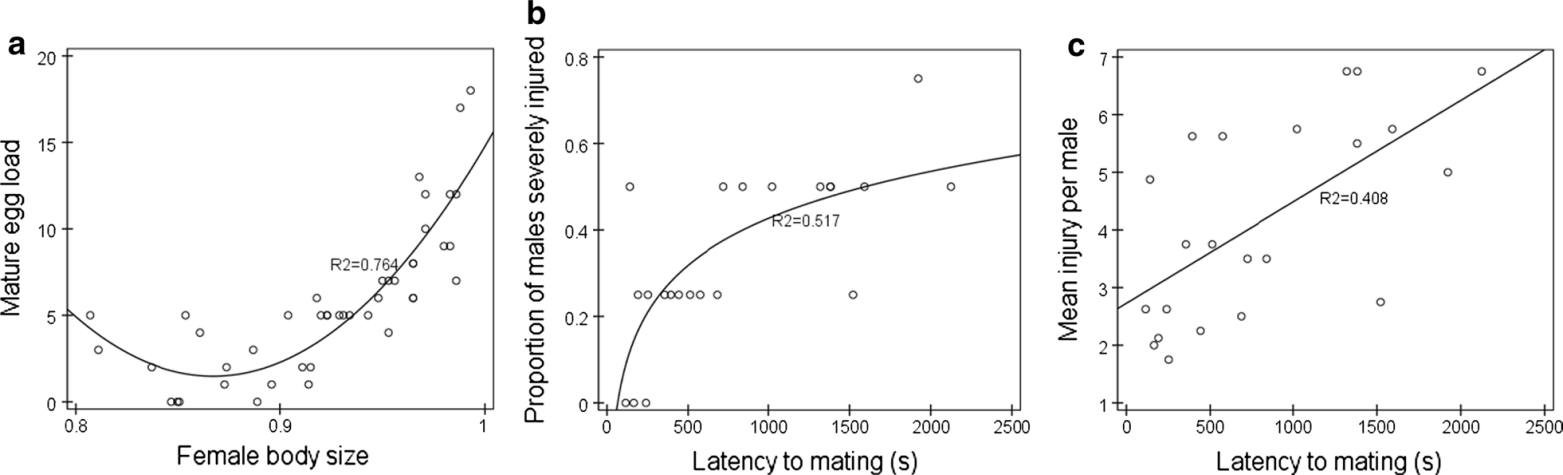
Relationships between female body size and mature egg load (**a**), between during of mating occurred and fighting intensity (**b**, **c**). The body size of parasitoid was measured by pleopod tibial length of adult

Females had on average 5.8 ± 0.64 mature eggs, and the quadratic equation of mature egg load (*y*) and female body size (*x*) was *y* = 570.38 − 1311.714*x* + 756.105*x*^2^ (Fig. 2a: *R*^2^ = 0.764, *p* < 0.001). Neither female body size nor egg load had a significant effect on the fighting intensity measured as the proportion of males injured (body size, *F* = 0.09, *df* = 1, *df*2 = 42, *p* > 0.05; egg load, *F* = 1.24, *df* = 1, *df*2 = 42, *p* > 0.05; interaction, *F* = 0.2, *df* = 1, *df*2 = 42, *p* > 0.05) or severely injured (body size, *F* = 0.01, *df* = 1, *df*2 = 42, *p* > 0.05; egg load, *F* = 0.09, *df* = 1, *df*2 = 42, *p* > 0.05; interaction, *F* = 0.49, *df* = 1, *df*2 = 42, *p* > 0.05) or the mean injury per male (body size, *F* = 0.96, *df* = 1, *df*2 = 42, *p* > 0.05; egg load, *F* = 0.93, *df* = 1, *df*2 = 42, *p* > 0.05; interaction, *F* = 4.74, *df* = 1, *df*2 = 42, *p* > 0.05).

#### Latency to mating

In the treatment in which males engaged in combat for a virgin female, the mating behaviour mainly (65.22%) occurred within 20 min after the experiment began. Except for the proportion of males injured (*F* = 0.03, *df* = 1, *df*2 = 21, *p* > 0.05), fighting intensity measured as proportion of males severely injured (*F* = 12.64, *df* = 1, *df*2 = 21, *p* < 0.001) and mean injury per male (*F* = 7.3, *df* = 1, *df*2 = 21, *p* < 0.05) in the treatment in which mating occurred earlier was significantly lower than that in the treatment in which mating occurred later. The logarithmic equation of proportion of males severely injured (*y*) and latency to mating (*x*) was *y* = 0.157ln*x* − 0.657 (Fig. 2b: *R*^*2*^ = 0.517, *p* < 0.01), and the linear equation of mean injury per male (*y*) and latency to mating (*x*) was y = 2.727 + 0.002*x* (Fig. 2c: *R*^*2*^ = 0.408, *p* < 0.01).

### Lethal combat for hidden females

**Fig. 3:**
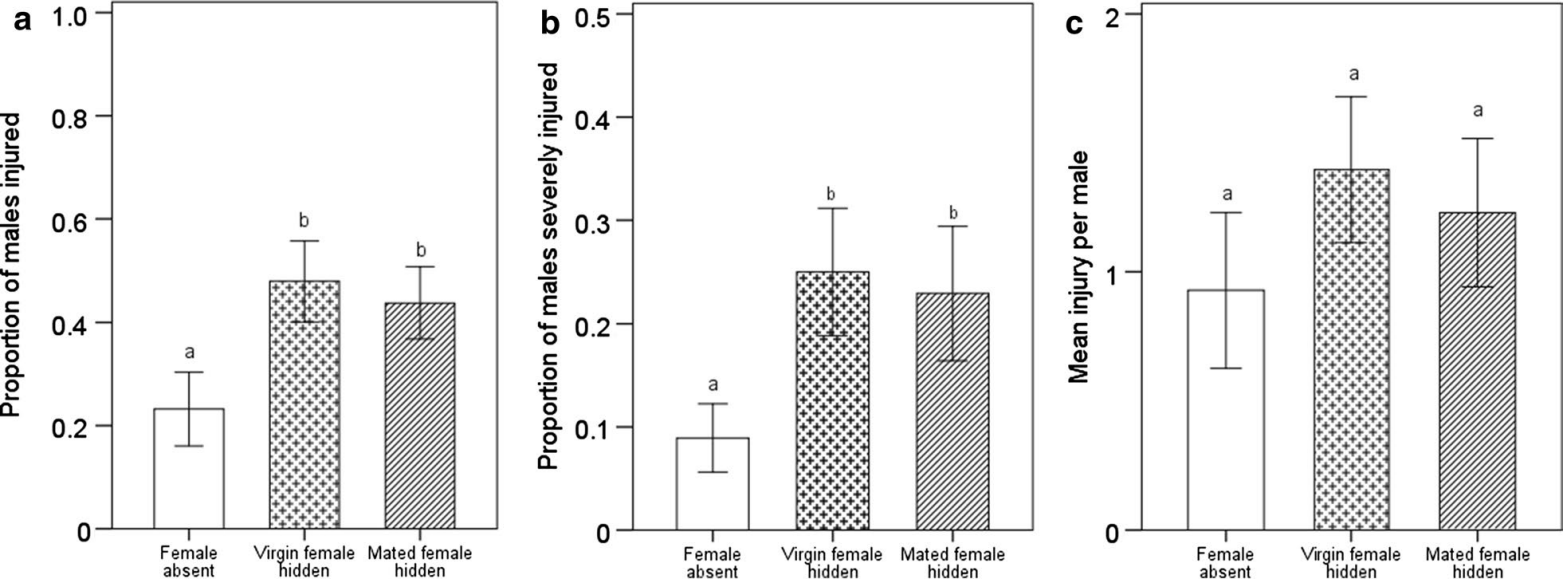
Proportions of males injured (**a**) and severely injured (**b**), and Mean injury per male (**c**) after combat for 3 h under conditions of female absence and a hidden female (virgin and mated females). Severely injured generally involved the loss of two legs or even more serious injuries. The same letter on the column indicates no significant difference, however, different letters indicate significant difference. The error bars indicate means ± standard errors

To explore whether males detected female mating status and adjusted their fighting behaviour accordingly based on chemical cues, we hid a female in the arena. Compared to males in the treatment without females, we found that males presented with a female (mated or virgin) hidden in their environment were significantly more likely to increase their fighting intensity measured as the proportions of males injured (Fig. 3a: virgin female: *F* = 5.75, *df* = 1, *df*2 = 24, *p* < 0.05; mated female: *F* = 4.37, *df* = 1, *df*2 = 24, *p* < 0.05) and severely injured (Fig. 3b: virgin female: *F* = 4.57, *df* = 1, *df*2 = 24, *p* < 0.05; mated female: *F* = 3.29, *df* = 1, *df*2 = 24, *p* < 0.05), except for the mean injury per male (Fig. 3c: virgin female: *F* = 3.35, *df* = 1, *df*2 = 24, *p* > 0.05; mated female: *F* = 1.76, *df* = 1, *df*2 = 24, *p* > 0.05). In the hidden experiment, the mating status of females did not significantly influence the fighting intensity measured as the proportions of males injured (*F* = 0.19, *df* = 1, *df*2 = 22, *p* > 0.05) and severely injured (*F* = 0.11, *df* = 1, *df*2 = 22, *p* > 0.05) and the mean injury per male (*F* = 0.17, *df* = 1, *df*2 = 22, *p* > 0.05).

## Discussion

In *A. disparis*, mates are a valuable resource for males, which significantly escalate their fighting behaviour when a female is present [28] (Fig. 1). In addition, our results showed that males also escalated their fighting behaviour when a female was hidden in the arena. This finding suggested that males were able to detect females in the environment based on a remotely detectable cue (e.g., sex pheromones) produced by females. Consistent with a number of empirical studies [11, 16, 21–24], *A. disparis* males adjusted their fighting behaviour according to resource value changed. Specifically, males fought more intensely to acquire a mating opportunity with a virgin female and fought less intensely when presented with a mated female (Fig. 1). For *A. disparis* males, variation in resource value was mainly associated with female mating status. Similar to many parasitoid wasp species [37], the *A. disparis* females exhibited the characteristics of monandry, in which females reject subsequent matings after they have already mated [31], which the benefit associated with male competition for mated females was reduced to zero. Escalated fighting behaviour is predicted to occur under conditions where the benefit of winning far outweighs the potential cost of conflict [2, 4]. However, there was relatively little benefit to be gained by fighting for access to mated females as the potential costs of doing so are high, which may have contributed to *A. disparis* males decreasing their fighting intensity for mated females. In addition, in the treatment with males engaging in combat for a virgin female, latency to mating had a significant effect on the final fighting intensity: the fighting intensity of the treatment in which mating occurred earlier was lower than that in the treatment in which mating occurred later. It is likely that when mating occurred earlier, males detected the change in resource value (i.e., female mating status) earlier and quickly adjusted their fighting behaviour, resulting in a decrease in the final fighting intensity compared to that observed when mating occurred later.

Before adjusting fighting behaviour, correctly estimating variation in the value of resources is necessary for competitors. How do *A. disparis* males estimate variation in the value of resources, i.e., female mating status? Here, we discuss two alternative possibilities. Mating often induces behavioural and physiological changes in female insects [38, 39]. Female behaviour may switch from that of being sexually receptive to that associated with the loss of receptivity [36]. The loss of receptivity may be accompanied by depletion of sex pheromone [40–42]. The amount of sex pheromone may be different between virgin and mated *A. disparis* females, which may be detected by males to estimate female status accordingly. However, expression of the pheromone biosynthesis-activating neuropeptide (PBAN) gene, which stimulates pheromone biosynthesis [43], was not significantly different between virgin and mated females (data from quantitative real-time PCR and transcriptional analyses [31]). In addition, in experiment two, in which females were hidden, whether a female had already mated did not significantly influence male fighting intensity (Fig. 3), which might also offer evidence that the substances produced by females (e.g., sex pheromone) do not differ significantly between virgin and mated females.

Alternatively, observational results showed that when males courted virgin females by knocking their antennae on the females’ antennae, the female usually remained still and responded by knocking on the opposite antennae; in contrast, when a male courted a mated female by knocking his antennae on the female’s antennae, the female tended to elude or flee. These behavioural differences may be easily detected by males and serve as evidence with which to estimate female status and resource value. Notably, males may be better able to clearly detect a female’s mating status after failing to court her multiple times than after a single attempt because after being rejected by a mated female one time, males sometimes still fan and run towards the female for courtship. In addition, these behavioural differences might well explain the phenomenon that although males decrease their fighting intensity for mated females with little potential benefit, the proportion of severely injured males in the presence of a female (17.86 ± 4.28%) is higher than that of males in the absence of a female. Males may require a period of time to clearly confirm the female’s mating status after failing to court her multiple times. Before doing so, males may engage in fatal fighting for valuable mating opportunities, resulting in increased fighting intensity. Currently, the reason for this phenomenon is not clear, and more studies, for example, on how many failed courtships are required for the male to determine the female’s mating status, need to be conducted to reveal this cue. Generally, it seems that although males are capable of discriminating between virgin and mated females, this ability might be not perfect.

In many studies, resource value is influenced by the fecundity of females, and individuals adjust their fighting behaviour in response to variation in the fecundity of females [20, 32]. However, this is unlikely to be the case in our study species, *A. disparis*. One explanation is that males may have no ability to accurately estimate the female’s fecundity (i.e., egg load). Many studies have suggested that individuals cannot estimate females’ fecundity directly and should refer to indirect information such as the amount of time he had been mating [19] and female body size [20, 32]. However, based on abundant empirical data, there is a positive relationship between female fecundity and body size in general [34], and specifically in *A. disparis*. In solitary and quasi-gregarious species, the size of the wasp is correlated with the size of the emerged host [44–46]. All *A. disparis* individuals in our study developed from hosts of the same species (i.e., an *A. pernyi* egg) and of similar sizes, and the body sizes of females were little variation in the current experiment, resulting in there was nothing for them to detect. Alternatively, as *A. disparis* males showed extreme fighting for mating opportunities [28], the question of whether they can be mated may be the most important aspect of the resource value of females, rather than other aspects of fecundity. Beside, as current experiment’s design that given no choice of female, they might fighting equally for what they can get. Further studies could set several females with different body size (i.e., fecundity) were present.

## Conclusions

In conclusion, females are valuable resources to *A. disparis* males, and the resource value is significantly influenced by mating status rather than by fecundity. *A. disparis* males have evolved extreme fighting behaviour and show a strong motivation to fight, engaging in extreme combat even in the absence of females [28] (also see Fig. 1). However, males do not always escalate fighting behaviour for the opportunity to mate with a female. Information on valuable resources (e.g., substances produced by females and changes in mating status) was collected and estimated by *A. disparis* males, which then adjusted their fighting behaviour accordingly and to some extent avoid fighting costs.

## Methods

### Insect cultures

*Anastatus disparis* colonies were first established from a population reared on *Lymantria dispar* egg masses collected in the wild, then subsequently maintained on *Antheraea pernyi* eggs [26, 27]. Twenty hosts were offered to a female for oviposition for 24 h at 26–28 °C. Then, we isolated the hosts individually in polyethylene tubes (height: 7.5 cm; diameter: 1 cm), the openings of which were covered with cotton balls to prevent any mating and fighting behaviour before the start of the experiment. The parasitized hosts were incubated at a temperature of 28 ± 0.5 °C, a relative humidity (RH) of 70 ± 5% and a photoperiod of 14 L:10 D. After approximately 18 days [27], females and males started to emerge and were collected daily.

### Experiment one: lethal combat for virgin and mated females

**Fig. 4:**
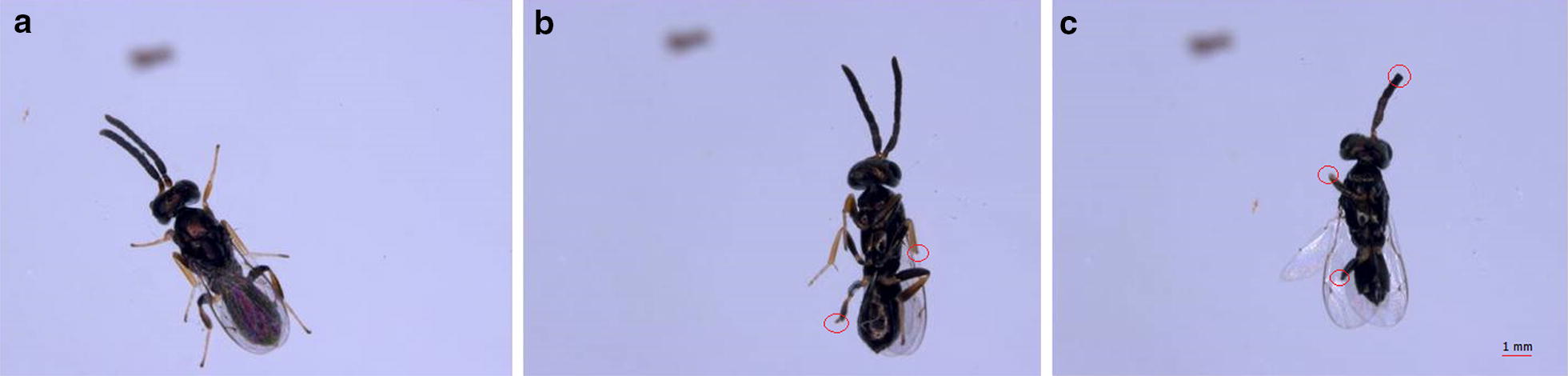
Healthy (**a**) and injured *A. disparis* male (**b**, **c**). Based on the degree of injured, injured male can be classified as either slight (**b**) or severe (**c**). Slight injuries were those where the male lost half of or a complete single leg or antenna; severely injured, which generally involved the loss of two legs or even more serious injuries

Extreme fighting behaviour for mating opportunities frequently occurs in *A. disparis* males. During the peak of eclosion (from 9:00 a.m. to 12:00 p.m.), male–male chasing and fighting near the emergence site are frequently observed; therefore, in this study, the fighting intensity of male combat lasting 3 h was tested. As the intensity of fighting increased with male density [28], fighting intensity of four males was tested for easier to observe fighting behaviour. Four 1-day-old males that enclosed from 9:00 a.m. to 10:00 a.m. were introduced into a cylindrical arena (height: 5 cm, diameter: 10 cm) containing a 2-day-old virgin (N = 23) or 2-day-old mated female (N = 21). Fighting experiments started at 10:00 a.m. and lasted 3 h. In the treatment consisting of male combat for a virgin female, we recorded the latency to mating. As mated females refuse to mate again, the latency to mating was not recorded. After 3 h, both females and males were removed. The female’s body size and fecundity were measured. Egg loads were represented as fecundity, which was measured as the number of mature eggs in the ovaries [33]. To explore whether males have the ability to adjust their fighting behavior in response to females with different fecundity, 2-day-old females were offered to males because newly emerged females have fewer mature eggs in the ovaries. The females were killed by freezing at − 80 °C, and their abdomens were then placed into a Petri dish with saline solution. We counted the number of mature eggs by dissecting the abdomens under a microscope using forceps (Leica M205A, Germany). The pleopod tibial length of adult *A. disparis* (a measure of parasitoid body size [47]) was measured under a microscope. All the males (dead, injured and healthy) were isolated individually in polyethylene tubes (height: 7.5 cm; diameter: 1 cm). During the 3 h of male combat, individuals rarely died; thus, we considered only the condition of injury, which could be classified as either severe or slight. Slight injuries were those where the male lost half of or a complete single leg or antenna (Fig. 4b). Immobile males were considered as severely injured, which generally involved the loss of two legs or even more serious injuries (e.g., loss all legs and antenna, Fig. 4c) [28]. The number of dead and injured males was recorded under a microscope; we also scored each visible male injury with the microscope according to a scale from 0 to 7 (e.g., the loss of an antenna scored 0.5 points) adapted from Murray [12, 13, 29, 48]. We then calculated the mean injury per wasp and the proportions of injured males in each arena.

To obtain mated females, newly emerged males were supplied to a virgin female (1 day old) for mating, and mating behaviour was observed. All adult females (virgin and mated females) were fed honey water (honey:water = 4:6) until the experiment began. The control treatment (N = 14) was similar, except that the four 1-day-old males were introduced into a cylindrical arena without a female.

### Experiment two: lethal combat for hidden females

Four 1-day-old males emerged from 9:00 a.m. to 10:00 a.m. were introduced into a cylindrical arena (height: 5 cm, diameter: 10 cm) containing a 2-day-old virgin (N = 12) or mated female (N = 12) hidden under a cover. Tissue paper was used as the cover material to allow ventilation. Fighting experiments started at 10:00 p.m. After 3 h, both males and females were removed. All the males (dead, injured and healthy) were isolated individually in polyethylene tubes (height: 7.5 cm; diameter: 1 cm). The number of dead and injured males was recorded using a microscope; we also scored each visible male injury with a microscope. We then calculated the mean injury per wasp and the proportions of injured males in each arena.

### Statistical analysis

All statistical analyses were performed using R software (version 2.14.1). Generalized linear mixed model (GLMM) was applied to analyse the fighting intensity data measured as the proportions of injured and severely injured males, and mean injury per male. When analyzing proportion data, model assumes binomial errors structure and uses a logit link function [49]. Nonproportion data of mean injury per male conformed to assumptions of GLMM analyses using normal error distributions. Proportion data often can be overdispersed that leaded to overestimation of significance. Overdispersion of data was tested by calculating the heterogeneity factor (HF), where HF > 1 data was scaled and model assumes quasi-binomial error distributions. In experiment one, we used the measures of fighting intensity as the response variables for each model, including mating status and female presence/absence as fixed effects, and the female’s body size and egg load and latency to mating as random effects. Interactions are presented only where significant at the level of *p* < 0.01; this criterion for significance is recommended when testing interactions [50]. In experiment two, fighting intensity was analyzed as the response variables, including mating status and hidden female presence/absence were included as fixed effects for each model. To check the relationships between female body size and mature egg load, and between latency to mating and fighting intensity, curve fitting were used to evaluate their regression.

## Data Availability

The data sets used and/or analysed during the current study are available from the corresponding author on reasonable request.

## Abbreviations

PBAN: pheromone biosynthesis-activating neuropeptide
RH: relative humidity
GLMM: generalized linear mixed model
